# Advanced glycation end-product receptor gene (RAGE) polymorphisms in patients with acute coronary syndrome – a case-control study in the Polish population

**DOI:** 10.1186/s12920-025-02215-3

**Published:** 2025-09-30

**Authors:** Klaudia Gutowska, Michał Ambroziak, Jakub Podraza, Monika Puzianowska-Kuźnicka, Krzysztof Czajkowski, Andrzej Budaj, Alina Kuryłowicz

**Affiliations:** 1 II Department of Obstetrics and Gynecology, Warsaw Medical University, Warsaw, Poland; 2 Doctoral School, Medical University of Warsaw, Zwirki i Wigury 81, Warsaw, Poland; 3 Department of Cardiology, Medical Centre of Postgraduate Education, Grochowski Hospital, Warsaw, Poland; 4 Department of Human Epigenetics, Mossakowski Medical Research Centre, Polish Academy of Sciences, Warsaw, Poland; 5 Deparment of Veterinary Medicine, Autonomous University of Barcelona, Barcelona, Spain; 6 Department of Geriatrics and Gerontology, Medical Centre of Postgraduate Education, Warsaw, Poland; 7 Department of General Medicine and Geriatric Cardiology, Medical Centre of Postgraduate Education, Warsaw, Poland

**Keywords:** Acute coronary syndrome (ACS), Coronary artery disease (CAD), Genetic predisposition, Receptor for advanced glycation end products (RAGE), Single nucleotide polymorphisms (SNP)

## Abstract

**Background:**

The development of coronary artery disease (CAD) is the result of complex interactions between environmental and genetic factors. While the former is well known, the genetic factors that predispose individuals to the development of CAD are still under investigation. The aim of our study was to investigate whether single nucleotide polymorphisms (SNPs) in the gene encoding the receptor for advanced glycation end products (*RAGE*), specifically rs2070600 G/A and rs184003 G/T, may determine predisposition to acute coronary syndrome (ACS) and severity of coronary artery disease (CAD) in the Polish population.

**Methods:**

Two *RAGE* SNPs were genotyped in 336 patients with a history of acute coronary syndrome (ACS): 175 < 50 years, 161 ≥ 50 years, and 160 ethnically, age- and sex-matched controls via the restriction fragment length polymorphism method. Allele frequencies were compared between groups via the chi^2^ test on a 2 × 2 contingency table. Genotype distribution was analyzed assuming three modes of inheritance: dominant, codominant, or recessive. The values of the variables between the study groups were compared using Student’s t-test or the Mann-Whitney *U* test, as appropriate.

**Results:**

For the rs184003 G/T polymorphism, the frequency of genotypes containing the T allele (GT + TT) was significantly greater in patients with a history of ACS than in healthy age- and sex-matched controls (28.87% vs. 11.25%, *p* < 0.0001, OR = 3.2 [95% CI: 1.86–5.52]). Moreover, individuals possessing this allele had lower high-density lipoprotein (HDL) cholesterol levels (mean 1.02 mmol/l vs. 1.14 mmol/l, *p* = 0.01) and higher median troponin I concentrations at the time of ACS (32.2 ng/ml vs. 24.4 ng/ml, *p* = 0.04). These genotypes were also significantly less common in patients with ACS before the age of 50 than in those diagnosed later (20.0% vs. 38.5%, *p* = 0.0002, OR = 0.4 [95%CI: 0.25–0.65]). In the case of rs2070600 G/A polymorphism, the genotype and allele frequencies were not significantly different between the study groups and subgroups.

**Conclusions:**

Our findings suggest that the rs184003 SNP in the *RAGE* gene may play a role in determining genetic susceptibility to CAD and ACS in the Polish population. However, they do not support the hypothesis that the studied SNPs impact the incidence of ACS at a young age.

**Supplementary Information:**

The online version contains supplementary material available at 10.1186/s12920-025-02215-3.

## Background

Persistent accumulation of vessel-occluding plaques in the intimal layer of medium- and large-sized arteries initiates damage to the endothelial cells responsible for the regulation of vascular integrity and barrier function [1, 2]. Under proinflammatory conditions, disrupted homeostasis promotes the restriction of blood flow, vascular stenosis, and cell hypoxia, eventually leading to atherosclerosis, which is one of the major causes of morbidity and mortality from cardiovascular disease [3, 4]. Accumulating evidence has shown that atherosclerosis is a complex disease involving multiple determinants, including genetic and modifiable environmental factors, as well as the impact of comorbidities, such as hypertension, diabetes mellitus, and dyslipidemia [5, 6]. However, the list of factors influencing the pathogenesis of this disease is still incomplete and warrants further study.

An increasing number of studies have suggested that advanced glycation end products (AGEs) contribute to diabetes-related complications [7]. AGEs are a heterogeneous group of macromolecular derivatives formed by an irreversible Maillard reaction that interact with the multiligand receptor for AGEs (RAGE), which is expressed in numerous tissues and cells, including vascular smooth cells [8, 9]. The binding of AGEs to RAGE leads to the activation of the inflammatory response through the activation of nuclear factor-β, oxidative stress, and proinflammatory cytokines [10, 11]. Both inflammatory and oxidative stress pathways play a role in atherosclerosis, which is initiated not only by hypertension or obesity but also by the deposition of excess sugars that promote the formation of AGEs [12]. Thus, the expression of RAGE on cells involved in plaque formation (e.g., endothelial cells, smooth muscle cells, and neutrophils) has been strongly demonstrated, particularly in diabetic patients [13]. However, subsequent studies have shown that RAGE also triggers thrombotic, profibrotic, and inflammatory responses in euglycemic patients [14]. Interestingly, the metalloproteinase-mediated proteolytic cleavage of RAGE results in the generation of a soluble isoform (sRAGE), and in addition, the direct alternative splicing of RAGE results in the generation of endogenous secretory RAGE (esRAGE), both of which interfere with membrane-bound RAGE [15]. Previous studies have shown that sRAGE has an anti-inflammatory and atheroprotective effect and that its high level slows the progression of intima-media thickness (IMT). It also reduces the risk of major coronary events and mortality [16]. Nevertheless, some studies have questioned the atheroprotective effect of sRAGE and associated it with the incidence of fatal and nonfatal cardiovascular disease [17, 18], whereas another study denied any relationship between sRAGE and carotid IMT and plaque [19]. These findings emphasize the important role of RAGE in atherosclerosis and related complications, but its causal role remains unclear.

Coronary artery disease (CAD) is a manifestation of atherosclerosis in the heart. Its genetic background is suggested by a familial and ethnic predisposition to the disease as well as a sex-related incidence. Both genome-wide association studies (GWASs) and candidate gene studies have identified a number of loci and single genes that may determine genetic predisposition to coronary atherosclerosis in a population-specific manner [20]. In our work, we hypothesized that the associations of sRAGE and/or esRAGE with the risk of coronary atherosclerosis may be determined by genetic variants in the *RAGE* gene. The aim of our study was therefore to assess the distribution of genotypes and alleles of two single nucleotide polymorphisms (SNPs) in *RAGE*, among Polish patients with acute coronary syndrome (ACS) stratified by age, and in healthy, age- and sex-matched controls.

## Materials and methods

### Study design

A case-control study was conducted, involving Polish patients with ACS and age- and sex-matched healthy control subjects.

### Study participants

The study population included individuals whose basic clinical characteristics were described previously [21]. The study group consisted of 175 unrelated patients (139 men and 36 women, 82.9% vs. 17.2%) aged less than 50 years (26–49 years, mean 43.5 years) who were admitted to the Department of Cardiology Grochowski Hospital in Warsaw for a first episode of acute coronary syndrome. ACS diagnosis was established on the basis of clinical symptoms (stenocardial pain), electrocardiography findings (non-ST-segment elevation myocardial infarction and ST-segment elevation myocardial infarction), and elevated troponin levels. Two control groups were included in the study. The first group comprised 160 ethnically matched unrelated healthy subjects aged 30–49 years (mean age 42 years), 105 men (65.6%) and 55 women (34.4%) with no history of CAD or diabetes who were recruited from healthy blood donors in collaboration with the regional blood center. The second control group consisted of 161 unrelated patients aged over 50 years (50–92 years, mean 65 years), 130 men (64.3%) and 41 women (35.7%) who were hospitalized in the Department of Cardiology Grochowski Hospital in Warsaw for a first episode of ACS (meeting the above criteria). All patients with ACS had percutaneous coronary intervention performed. Coronary angiography was recorded in digital form and assessed for ongoing study by an independent invasive cardiologist blinded to the patient history and ECG and echocardiographic data. CAD extent was assessed using SYNTAX and EXTENT scores [22, 23]. The baseline clinical characteristics of both ACS groups are summarized in Supplementary Table 1.

Data on comorbidities, including current treatment, were collected via patient questionnaires (Supplementary File 1) and physical examinations on admission. Hypertension was assessed on the basis of history and treatment but also on the basis of the mean value of two measurements of systolic (SBP) and diastolic (DBP) blood pressure performed at 5-minute intervals. Hypertension was defined as values ≥ 140 mmHg SBP and/or ≥ 90 mmHg DBP according to the European Society of Hypertension, and European Society of Cardiology guidelines [24]. Diabetes was assessed by history and treatment or by a fasting plasma glucose level ≥ 126 mg/dl (7.0 mmol/l) or ≥ 200 mg/dl (11.1 mmol/l) in an oral glucose tolerance test or an HbA1c value ≥ 6.5% according to the European Association for Study of Diabetes and American Diabetes Association guidelines [25]. Data on depression and smoking status, including duration and intensity (number of cigarettes per day), were collected during a patient interview. Body mass index (BMI) was calculated as weight (kg)/height (m^2^). Blood samples were taken on admission (to determine admission glucose) and the following morning (to determine other parameters). Biochemical analyses, including glucose, total cholesterol, high-density lipoprotein (HDL) and low-density lipoprotein (LDL) cholesterol, and plasma triglyceride (TG) levels, were performed on fasting blood samples (except for glucose at enrollment) via standard enzymatic methods and COBAS INTEGRA 800 equipment (Roche Diagnostics GmbH).

The study adhered to the tenets of the Declaration of Helsinki, the research program was approved by the local ethical committees, and written informed consent was obtained from all the participants.

### DNA extraction and genotyping

Genomic DNA was isolated from peripheral blood mononuclear cells via the salting-out method [26]. Single nucleotide polymorphisms in *RAGE* (GenBank NC_000006.12:32183665) were generated via polymerase chain reaction (PCR) amplification followed by digestion with restriction enzymes (restriction fragment length polymorphism – RFLP – method). Briefly, fragments of *RAGE* were amplified via PCR. The PCR conditions were as follows: initial denaturation at 94 °C for 5 min, followed by 35 cycles of 94 °C for 30 s, annealing at 62 °C for 30 s, extension at 72 °C for 30 s, and a final step at 72 °C for 5 min. Each 12.5 µl reaction contained 50 ng of DNA, 2.0 mM MgCl_2_, 10 pmol of each primer, 0.25 mM deoxynucleoside triphosphate and 1 unit of Taq polymerase (Invitrogen Carlsbad, USA) in the corresponding buffer. A total of 2.5 µl of the PCR product was digested with 1 unit of the proper restriction enzyme (Fermentas, Waltham, Massachusetts, USA) at 37 °C for 3 h. The reaction conditions (including primer sequences, annealing temperatures, and restriction enzymes) are listed in Table 1. The obtained restriction fragments were visualized on a 3% agarose gel (Merck, Darmstadt, Germany). To confirm the accuracy of the method employed, randomly selected samples from 10% of subjects were analyzed via direct sequencing by the external service (Laboratory of DNA sequencing and synthesis, Institute of Biochemistry and Biophysics, Polish Academy of Sciences, Warsaw Poland).

**Table 1:** Genotyping conditions

SNP	primers	annealing	product	restriction enzyme	alleles
**rs184003 G/T** **(1704G/T)**	F: 5’GCCCTCTCCTCAAATCCACT3’	62^◦^C	275 bp	BfaI	T 156, 119 bp G 156, 77, 42 bp
	R: 5’TGGTCCTGAGGCCCTATCT3’				
**rs2070600 G/A**	F: 5’GAAGGTCCTGTCTCCCCAG3’	62^◦^C	272 bp	AluI	G 272 bp A 213, 59 bp
	R: 5’AATGAGGCCAGTGGAAGTCA3’				

*SNP* Single-nucleotide polymorphism, *F* Forward, *R* Reverse, *BP* Base pairs

### Statistical analysis

Allele frequencies were compared between groups via the chi^2^ test on a 2 × 2 contingency table via the Statistica software package (StatSoft Inc., Tulsa, OK, USA). Genotype distribution was analyzed assuming three modes of inheritance: dominant, codominant, or recessive. For each model, the p-value for association was calculated. The appropriateness of the model was also assessed via the chi^2^ test. These calculations were performed via the Web-Assotest program (available at http://www.ekstroem.com/assotest/assotest.html). p values less than 0.05 were considered significant. Conservative Bonferroni correction for multiple testing was applied. The values of the variables between the study groups were compared via Student’s t-test or the Mann‒Whitney *U* test when appropriate. Assessment of normality of the distribution was performed with the Kolmogorov–Smirnov test.

## Results

As the distributions of the genotypes and alleles of the studied polymorphisms did not differ between the female and male study participants, all the analyses were performed together without stratification by sex. For both the polymorphisms studied and for all the groups and subgroups tested, the distributions of genotypes and alleles were consistent with Hardy-Weinberg equilibrium (*p* > 0.05).

### The genotype and allele distributions of RAGE polymorphisms differ between ACS patients and age- and sex-matched controls

We started our analysis by comparing the frequencies of the different genotypes and alleles of the investigated polymorphisms between the study groups (Table 2).

For the rs184003 G/T polymorphism, the frequency of genotypes containing the T allele (GT + TT) was significantly greater in patients with a history of ACS than in healthy age- and sex-matched controls (28.87% vs. 11.25%, *p* < 0.0001, OR = 3.2 [95% CI: 1.86–5.52]). Consistently, allele T was more common in ACS patients than in the healthy age- and sex-matched group (15.33% vs. 5.94%, *p* < 0.0001, OR = 2.86 [95%CI: 1.72–4.47]).

The situation was different for the rs2070600 G/A polymorphism: genotype and allele frequencies were not significantly different between the study groups (after Bonferroni correction).

**Table 2:** Distribution of genotypes and alleles of *RAGE* polymorphisms in the study groups

		ACS < 50		Healthy controls			p* OR [95% CI]	ACS ≥ 50				p** OR [95% CI]	All ACS		p*** OR [95% CI]
rs184003		%	%	*N*	%	%		*N*	*N*	%	%		*N*	%	
Genotypes		175		160				161					336		
GG		140	80.0	142	88.75			99		61.50			239	71.13	
GT		33	18.85	17	10.65			58		36.02			91	27.08	
TT		2	1.15	1	0.60			4		2.48			6	1.79	
GT + TT vs. GG							*p* = 0.027^ 1.97 [1.3–3.65]					*p* = 0.0002 0.4 [0.25–0.65]			*p* < 0.0001 3.2 [1.86–5.52]
GG vs. TT							*p* = 0.499					*p* = 0.405			*p* = 0.268
GT + GG vs. TT							*p* = 0.611					*p* = 0.350			*p* = 0.271
Alleles															
G		313	89.43	301	94.06		*p* = 0.03^	256		79.50		*p* < 0.0001 0.24 [0.09–0.75]	569	84.67	*p* < 0.0001
T		37	10.57	19	5.94		1.87 [1.08–3.32]	66		20.50			103	15.33	2.86 [1.72–4.71]
rs2070600		N	%	N	%			N		%			N	%	
Genotypes		175		160				161					336		
GG		150	85.71	123	76.87			121		75.15			271	80.65	
GA		24	13.71	32	20.0			35		21.74			59	17.56	
AA		1	0.58	5	3.13			5		3.11			6	1.79	
GA + AA vs. GG							*p* = 0.052					*p* = 0.081			*p* = 0.334
GG vs. AA							*p* = 0.097					*p* = 0.095			*p* = 0.244
GA + GG vs. AA							*p* = 0.087					*p* = 0.089			*p* = 0.357
Alleles															
G		324	92.57	278	86.87		*p* = 0.023^	277		86.02		*p* = 0.02^	601	89.43	*p* = 0.140
A		26	7.43	42	13.13		0.53 [0.31–0.89]	45		13.98		0.49 [0.29–0.82]	71	10.56	

*ACS* Acute coronary syndrome, *CI C*onfidence, *N* Number, *OR* Odds ratio, *SNP* Single-nucleotide polymorphism

^ *p-*values non-significant after Bonferroni correction

*calculations performed for ACS < 50 vs. Healthy controls

**calculations performed for ACS < 50 vs. ACS ≥ 50

***calculations performed for All ACS vs. Healthy controls

### Genotype and allele distributions of RAGE polymorphisms differ between patients with ACS before and after age 50

In subsequent analyses, we wanted to test whether the presence of any of the polymorphic variants studied could predispose the participants in our study to ACS at a younger age. To do this, we compared the frequencies of individual genotypes and alleles in patients diagnosed with ACS before and after the age of 50 and related them to the frequencies observed in the healthy age- and sex-matched group.

For the rs184003 G/T polymorphism, the frequency of genotypes differed between patients diagnosed with ACS before and after the age of 50. Genotypes containing the polymorphic T allele (GT and TT) were significantly less common in patients with ACS before the age of 50 than in those diagnosed later (20.0% vs. 38.5%, *p* = 0.0002, OR = 0.4 [95%CI: 0.25–0.65]). The frequency of genotypes containing the polymorphic T allele was greater in the group of patients diagnosed with ACS before the age of 50 years than in the group of healthy controls (20% vs. 11.25%), but this difference was not statistically significant after Bonferroni correction (*p* > 0.05). In the group of patients with ACS diagnosed after the age of 50, the frequency of genotypes containing the polymorphic T allele was significantly greater than that in healthy controls (38.5% vs. 11.25%, *p* < 0.0001, OR = 0.24 [95%CI: 0.09–0.75]).

In the case of the rs2070600 G/A polymorphism, we observed that although the frequency of genotypes did not differ between all ACS patients and controls, genotypes containing the A allele (GA + AA) were less common in patients who had ACS before the age of 50 (14.29% vs. 24.85%), but this difference was not statistically significant after correction.

### Distribution of RAGE genotypes and severity of coronary artery disease

In this study, we explored whether *RAGE* genotype distribution differs in patients with different severity of coronary artery stenosis, too. The dataset included measures of CAD severity, specifically the degree of stenosis in individual coronary arteries, along with the mean and median stenosis values.

Among younger patients, the median degree of stenosis (defined as average across vessels) was 25.75%. In older patients, the median degree of stenosis was 49.27%. These observations may suggest a potential trend in which certain genotypes may be associated with a more advanced form of CAD.

However, we observed no significant correlation between the degree of coronary artery stenosis and the genotypes of the RAGE polymorphisms studied in the examined groups of CAD patients. In patients diagnosed with ACS under the age of 50, the median degree of coronary artery stenosis was 22.83% in carriers of the rs184003 T polymorphism (GT and TT), compared to 26.35% in those with the GG genotype. For participants who experienced ACS at 50 years of age or older, the median degree of coronary artery stenosis was 47.15% for GT and TT genotype carriers, versus 50.25% for GG genotype carriers. For the rs2070600 SNP, the median degree of coronary artery stenosis was 27.30% in younger patients with genotypes containing the A allele (GA + AA), compared to 25.25% in GG genotype carriers. In older participants, these values were 48.15% and 52.55%, respectively.

### Patients with the polymorphic rs184003 T allele have different clinical characteristics from GG homozygotes

To test whether the polymorphisms studied can modulate the clinical course of ACS, we examined the distribution of biochemical and clinical parameters between study participants, stratified by the presence of polymorphic alleles of the genetic variants studied.

We observed that patients carrying the T allele of the rs184003 polymorphism (GT + TT genotypes) had slightly lower HDL cholesterol levels (mean 1.02 mmol/l vs. 1.14 mmol/l, *p* = 0.01) than those homozygous for the G allele. These patients also had significantly higher median troponin I levels at the time of ACS (32.2 ng/ml vs. 24.4 ng/ml, *p* = 0.04) than did the carriers of the GG genotype. However, we did not observe statistically significant differences in total cholesterol, LDL cholesterol, triglycerides, glucose on admission, fasting glucose, or renal function parameters between study participants carrying different variants of the rs184003 G/T polymorphism.

For the rs2070600 G/A polymorphism, we found no statistically significant differences in biochemical parameters between patient groups stratified according to the presence of the polymorphic A allele (AA + GA vs. GG).

We also tested whether the genotype frequencies of the polymorphisms studied differed among patients with diabetes, a family history of cardiovascular disease, and those who had suffered an ST-elevation myocardial infarction, but we detected no statistically significant differences, probably due to the limited number of study participants that negatively impacted the power of the performed analysis.

## Discussion

The development of CAD is the result of complex interactions between environmental and genetic factors. While the former is well known and includes, among others, older age, smoking, and comorbidities such as diabetes, hypercholesterolemia, or poorly controlled blood pressure, the genetic factors that predispose individuals to the development of CAD are still under investigation [20]. The aim of our case-control study was to investigate whether single nucleotide substitution polymorphisms (rs184003 G/T and rs2070600 G/A) in the *RAGE* gene may determine predisposition to CAD in the Polish population.

The choice of candidate gene and polymorphisms studied was not accidental. As mentioned above, RAGE has a well-established role in the pathogenesis of CAD [11], while its pharmacological blockage or genetic deletion results in significant suppression of atherosclerosis progression [27]. The *RAGE* gene is located on chromosome 6p21.3 (at a locus involved in inflammatory and immune responses and tissue compatibility complex III) and is overexpressed in atherosclerotic plaques [28]. It has also been identified as a likely candidate gene associated with vascular and neurological complications such as atherosclerosis, CAD, ischemic stroke, and Alzheimer’s disease [29]. More than 50 polymorphic variants have been identified in the *RAGE* region, and association studies have linked them to the risk of a range of diseases, including cardiovascular, metabolic, and cancer [30]. Of these, the rs2070600 G/A SNP in exon 3 resulting in a missense mutation and glycine (G)-to-serine (S) substitution at position 82 of the amino acid chain is of special interest since it is located in the putative site of ligand binding, which could influence the AGE/RAGE interaction. The polymorphic A variant has potentially increased ligand binding affinity, which may predispose patients to or aggravate atherosclerosis [31]. In addition, the rs2070600 SNP has been reported to decrease the proteolysis of RAGE, thus affecting serum levels of sRAGE. Carriers of the GG genotype have been reported to have higher plasma levels of sRAGE than GA heterozygotes and AA homozygotes [32]. The rs2070600 SNP was subsequently associated with increased stimulation of RAGE by its ligands and the potential of a proinflammatory pathway [33]. However, data from studies on the association of this polymorphism with the risk of CAD in humans are inconclusive. Notably, the effect of the rs2070600 SNP on the development of coronary atherosclerosis may be population-specific [34]. In our study, the observed frequency of the polymorphic A allele in the healthy control group (13.98%) was similar to that described in other Caucasian populations [34]. In contrast, in the CAD groups, the prevalence of the A allele and GA/AA genotypes was lower than that in healthy controls, especially in those who had ACS at the age of 50 years or older. This finding would be interesting because a lower frequency of the polymorphic rs2070600 variant should theoretically result in less inflammation induced by the AGE-RAGE pathway and lower levels of sRAGE, which protects against the development of atherosclerosis. However, the difference in the frequency of polymorphic alleles and genotypes observed between the study groups did not reach statistical significance.

The second SNP studied in the *RAGE* is rs184003, a G/T substitution in introns 7/8 (known as 1704G/T). Much less is known about the biological significance of this SNP and its association with human disease. Since introns contain many functional elements, including intron-splicing enhancers and silencers that regulate alternative splicing, trans-splicing elements, and other regulatory elements, intron mutations can have potential consequences similar to those in the coding parts of genes. Polymorphisms in introns may also confer disease susceptibility or otherwise modulate the genotype-phenotype relationship [35]. The rs184003 polymorphic allele T has subsequently been found to be associated with a number of diseases and conditions, including insulin resistance and diabetes, high blood pressure, and different types of cancer [29]. The pathophysiological relevance of this polymorphism is not fully understood, but in a study of patients with gastric cancer, the presence of the rs184003 T allele was associated with higher sRAGE levels as compared with the wild-type homozygotes [36]. In turn in patients with schizophrenia, rs184003 was found to be in linkage disequilibrium with two other SNPs, rs2071288 and rs17846798, creating a specific haplotype whose carriers had lower serum esRAGE concentrations. esRAGE is an isoform of RAGE that lacks transmembrane and signaling domains and is thought to act as a decoy receptor, binding AGEs and reducing AGE-RAGE interactions and the activity of related intracellular signaling pathways [37]. This concept appears to be consistent with observational data suggesting that individuals with the T allele have significantly lower plasma levels of antioxidants, such as total carotenoids, lutein, lycopene, and tocopherol, than individuals with the 1704G allele. This suggests a potential role for the G1704T polymorphism in oxidative stress [38].

In our study, the observed frequency of this allele in the healthy age- and sex-matched group reflected that reported in other European Caucasian populations (4.9–6.8%) [29, 39]. However, in patients with CAD, the polymorphic genotypes TT and GT were found significantly more frequently. A similar trend indicating a link between the T allele with CAD risk was observed in a study of the Chinese population [40]. In this study, the frequency of the T allele in patients with CAD was significantly higher compared to healthy controls (20.62% vs. 17.59%), while the presence of the polymorphic genotypes (GT + TT) was associated with a risk of coronary artery atherosclerosis (OR = 2.39). However, it should be noted that, although this study was much larger than ours, it included patients with CAD (more than 50% stenosis in at least one of the three major coronary arteries or major branches), but not with a history of ACS. To our knowledge, our study is the first to describe a potential association between the rs184003 polymorphism and the occurrence of ACS in European Caucasian populations.

From a pathophysiological point of view, the higher prevalence of a genetic variant potentially associated with increased inflammation and oxidative stress in a group of patients with symptomatic CAD seems reasonable. Moreover, we observed that carriers of the polymorphic rs184003 T allele had significantly higher mean troponin I levels at the time of ACS than did those homozygous for the wild-type G allele and tended toward lower HDL-C concentrations. However, in the studied population, the distribution of the investigated genotypes in the subgroups stratified by the myocardial infarction type or severity of coronary artery stenosis did not differ significantly.

This research highlights a potential link between CAD severity and genetics. Our initial findings suggest that certain genotypes may affect the degree of artery stenosis, whereas previous studies have focused mostly on genetic contributions to the early onset of myocardial infarction [41, 42]. This finding is consistent with other studies that revealed genetic factors that influence the development of atherosclerosis without reference to acute coronary events [43]. However, our results should be interpreted cautiously, as the observed differences were not statistically significant.

To identify factors predisposing patients to the development of CAD and the incidence of ACS at a young age, we compared the frequencies of the genotypes studied in patients stratified by age. However, in our study, the frequency of polymorphic genotypes was greater in individuals diagnosed with ACS in their 50 s than in those in younger age groups. This finding was surprising since one would expect the pathogenic allele to be more common in younger patients with ACS, i.e., those younger than 50 years of age. These results therefore suggest a potential impact of rs184003 on the prevalence of CAD and ACS in the Polish population; however, this polymorphism does not appear to increase the risk of ACS at a young age.

We are aware of the potential limitations of this study. The most important limitation is the relatively small size of the study groups, which obviously affects, especially in the subgroup analysis, the power of the results obtained. On the other hand, the careful selection of patients ensures that they are well characterized and that we have complete clinical data that can be used for future meta-analyses. In addition, by analyzing only two of the more than fifty SNPs in the *RAGE* gene, we may have missed other potentially pathogenic variants. Furthermore, our lack of ability to measure sRAGE/esRAGE levels in the patient’s serum prevents us from assessing the extent to which the studied *RAGE* gene variants influence receptor concentrations in the bloodstream, and consequently the risk of ACS.

When discussing the potential limitations of our study, we must also acknowledge that when referring to the control group as ‘healthy, age- and gender-matched controls’, we cannot guarantee that these individuals did not have hidden coronary artery disease (CAD). According to applicable Polish legislation, only individuals who are healthy and show no signs or symptoms of disease (including chronic conditions that increase the risk of atherosclerosis) are permitted to donate blood. Additionally, only non-smokers were eligible to participate in this study. However, these criteria could not guarantee that the control group did not have latent coronary artery disease. Moreover, we did not collect information on donors’ family history of coronary artery disease.

Among the factors that may have influenced the results of this study, it should be noted that the ‘healthy control group’ was selected as a reference point for patients who had experienced ACS before the age of 50. Consequently, the entire study group of patients with a history of ACS (including individuals both under and over 50 years of age) was significantly older than the ‘healthy control group’.

Finally, the definition of ACS in young patients has not been precisely determined. In the literature, this term is used in studies describing individuals who experienced ACS at ages below 45, 30, and even 20 years. To ensure a similar number of participants in the groups being compared in our study, we set this threshold at under 50 years. Setting a different threshold (e.g. at age 45 or 55) would undoubtedly affect the results. All these above-mentioned factors could have influenced the results of our study.

## Conclusions

In conclusion, we found a higher frequency of the T allele of the *RAGE* rs184003 polymorphism among Polish patients with ACS, while the distribution of the rs2071288 SNP alleles and genotypes did not differ in patients with ACS and in the healthy age- and sex-matched group. However, our findings do not suggest a link between the studied SNPs and the occurrence of ACS at a young age. Further, well-designed studies with large sample sizes are needed to validate the present results.

## Supplementary Information

Supplementary material 1.

Supplementary material 2.

## Data Availability

The datasets generated and/or analyzed during the current study are available in the repository available at: https://figshare.com/articles/dataset/RAGE_rs184003_rs2070600_genotyping_xlsx/28887671?file=54028739.

## Abbreviations

ACS: Acute coronary syndrome
AGEs: Advanced glycation end products
BMI: Body mass index
CAD: Coronary artery disease
DBP: Diastolic blood pressure
esRAGE: Endogenous secretory receptor for advanced glycation end products
GWAS: Genome-wide association studies
HDL: High-density lipoprotein cholesterol
IMT: Intima-media thickness
LDL: Low-density lipoprotein cholesterol
OR: Odds ratio
PCR: Polymerase chain reaction
RAGE: Receptor for advanced glycation end products
RAGE: The gene for the receptor for advanced glycation end products
RFLP: Restriction fragment length polymorphism
SBP: Systolic blood pressure
SNP: Single nucleotide polymorphism
sRAGE: Soluble isoform of the receptor for advanced glycation end products
TG: Triglycerides
