# Supplementary material for: Advanced glycation end-product receptor gene (RAGE) polymorphisms in patients with acute coronary syndrome – a case-control study in the Polish population
Source: BMC Med Genomics. 2025 Sep 30;18:143. doi: 10.1186/s12920-025-02215-3 (PMC12482394; doi:10.1186/s12920-025-02215-3)
Supplement: Supplementary file 1 — Supplementary material 1. [file 12920_2025_2215_MOESM1_ESM.docx]

**Supplementary Table 1.** Baseline clinical and biochemical characteristics of patients with acute coronary syndrome (ACS).

|  | **All patients** | **< 50 years** | **≥ 50 years** | P* |
| --- | --- | --- | --- | --- |
|  | N=336 | N=175 | N=161 |  |
| **Female: Male (N)** | 67 : 269 | 36 : 139 | 31 : 130 | 0.763 |
| **Age (years, mean ± SD)** | 51.6 ± 11.3 | 44.2 ± 4.2 | 61.8 ± 12.1 | < 0.0001 |
| **Marital status (%)** |  |  |  |  |
| Married | 77.4 | 80.3 | 72.3 | 0.087 |
| Single | 10.7 | 14.1 | 7.3 | 0.052 |
| Divorced | 1.8 | 0.0 | 3.7 | 0.053 |
| Widowed | 10.1 | 5.6 | 14.7 | 0.01 |
| **Education (%)** |  |  |  |  |
| Primary | 8.9 | 6.9 | 11.1 | 0.165 |
| Vocational | 31.3 | 34.5 | 27.9 | 0.211 |
| Secondary | 41.3 | 36.5 | 46.3 | 0.054 |
| Higher | 18.5 | 22.1 | 14.7 | 0.083 |
| **Employment (%)** |  |  |  |  |
| Unemployed | 2.6 | 2.9 | 2.4 | 0.832 |
| Sedentary work | 43.0 | 47.5 | 38.3 | 0.099 |
| Physical work | 30.7 | 36.7 | 24.5 | 0.03 |
| Retirement | 23.7 | 12.9 | 34.8 | 0.0003 |
| **Family history of CVD** | 53.6 | 62.1 | 44.5 | 0.003 |
| **Smoking** (%) | 80.6 | 86.2 | 69.1 | 0.0005 |
| **Comorbidities (%)** |  |  |  |  |
| Hypertension | 62.5 | 59.3 | 66.4 | 0.197 |
| Type 2 diabetes | 30.4 | 24.2 | 36.4 | 0.02 |
| Depression | 8.3 | 8.1 | 8.5 | 0.871 |
| **Prior treatment (%)** |  |  |  |  |
| ASA | 22.3 | 17.0 | 27.7 | 0.03 |
| Statin | 27.6 | 22.7 | 32.1 | 0.02 |
| β-blocker | 38.3 | 27.7 | 49.6 | 0.0002 |
| ACEi/ARB | 53.7 | 39.7 | 44.4 | 0.385 |
| **STEMI (%)** | 67.2 | 72.0 | 62.9 | 0.04 |
| **BMI (kg/m^2^)** | 28.0 ± 4.1 | 28.6 ± 4.1 | 27.2 ± 3.8 | 0.002 |
| **Biochemical parameters** |  |  |  |  |
| Total cholesterol (mmol/l, mean ± SD) | 5.25 ± 1.2 | 5.5 ± 1.0 | 5.0 ± 1.3 | 0.002 |
| LDL-cholesterol (mmol/l, mean ± SD) | 3.4 ± 1.1 | 3.5 ± 1.0 | 3.2 ± 1.1 | 0.07 |
| HDL-cholesterol (mmol/l, mean ± SD) | 1.1 ± 0.3 | 1.0 ± 0.2 | 1.1 ± 0.25 | 0.03 |
| Triglycerides (mmol/l, mean ± SD) | 1.6 ± 0.8 | 1.9 ± 0.9 | 1.4 ± 0.6 | < 0.0001 |
| Glucose on admission  (mmol/l, median [25-75 quartile]) | 7.0  [5.8-8.5] | 6.4  [5.6-7.8] | 7.3  [6.3-8.9] | 0.0004 |
| FPG  (mmol/l, median [25-75 quartile]) | 5.4  [4.9-6.1] | 5.4  [4.9-5.6] | 5.5  [4.9-6.6] | 0.175 |
| Creatinine  (μmol/l, median [25-75 quartile]) | 97.3  [79.6-106.1] | 94.6  [78.7-106.1] | 97.3  [79.6-106.1] | 0.115 |
| Troponin I  (ng/ml, median [25-75 quartile]) | 22.1  [7.15-45.8] | 26.95  [8.36-50.86] | 27.76  [5.63-39.6] | 0.01 |

* Comparison of groups < 50 and **≥** 50 years

ACEi – angiotensin-converting enzyme inhibitor, ARB – angiotensin receptor blockers, ASA – acetylsalicylic acid, BMI – body mass index, CVD – cardiovascular diseases, FPG – fasting plasma glucose, HDL – high density lipoprotein, LDL – low density lipoprotein, N – number, NS – non significant, SD – standard deviation, STEMI – ST-segment elevation myocardial infarction.
