# Supplementary figures and images for: Advanced glycation end-product receptor gene (RAGE) polymorphisms in patients with acute coronary syndrome – a case-control study in the Polish population
Source: BMC Med Genomics. 2025 Sep 30;18:143. doi: 10.1186/s12920-025-02215-3 (PMC12482394; doi:10.1186/s12920-025-02215-3)

Supplementary Figure 1

1. rs184003


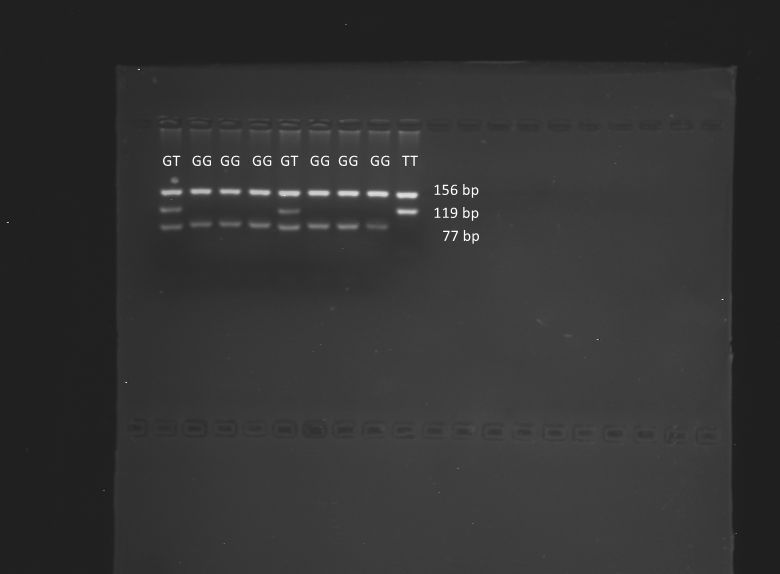


1. rs2070600


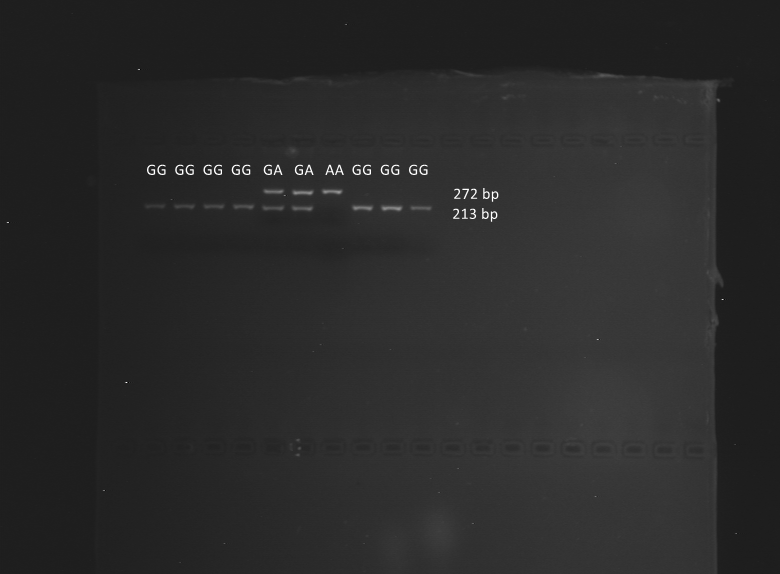

Supplement: Supplementary file 2 — Supplementary material 2. [file 12920_2025_2215_MOESM2_ESM.docx]
